# Anticancer and apoptotic effects on cell proliferation of diosgenin isolated from *Costus speciosus* (Koen.) Sm

**DOI:** 10.1186/s12906-015-0836-8

**Published:** 2015-09-02

**Authors:** Samy Selim, Soad Al Jaouni

**Affiliations:** Department of Clinical Laboratory Sciences, College of Applied Medical Sciences, Aljouf University, Sakaka, P.O. 2014 Saudi Arabia; Scientific Chair of YAJ Prophetic Medicine Application, College of Medicine, King Abdulaziz University, P.O. Box 80215, Jeddah, 21589 Saudi Arabia; Microbiology and Botany Department, Faculty of Science, Suez Canal University, Ismailia, P.O. 41522 Egypt

**Keywords:** Anticancer, Apoptotic, Diosgenin, *Costus speciosus* (Koen.) Sm

## Abstract

**Background:**

Diosgenin, a naturally occurring steroid saponin found abundantly in *C. speciosus*, is a well-known precursor of various synthetic steroidal drugs that are extensively used in the pharmaceutical industry.

**Methods:**

The present study was conducted to evaluate the *in vitro* anticancer and apoptotic effects on cell proliferation of diosgenin isolated from *C. speciosus* (Koen.) Sm.

**Results:**

The results indicated that the treatment of HepG2 cells with the sample resulted in a cytotoxic effect as concluded from the IC_50_ value 32.62 μg/ml, while the treatment of HepG2 cells with paclitaxel, a known anti-cancer drug, resulted in an IC_50_ value of 0.48 μg/ml. The treatment of MCF-7 cells with the tested sample resulted in high inhibition in the cell viability, and resulted in an IC_50_ value of 11.03 μg/ml, while the treatment of MCF-7 cells with paclitaxel resulted in an IC_50_ value of 0.61 μg/ml. The levels of DR4 and caspase-3 were significantly increased (*P* < 0.01) in MCF-7 cells treated with the tested sample compared to untreated cells and possessed a similar activity of paclitaxel in DR4 induction but lower induction in caspase-3. On the other hand the treatment of macrophages or lymphocytes with diosgenin (250 μg/ml) resulted in an induction in the cell proliferation up to 3.2-fold and 2.1-fold of control, respectively.

**Conclusions:**

The results presented here may suggest that diosgenin isolated from *C. speciosus* possess anticancer and apoptotic effects on cell proliferation, and therefore, can be used as pharmaceuticals drugs.

## Background

**Fig. 1:**
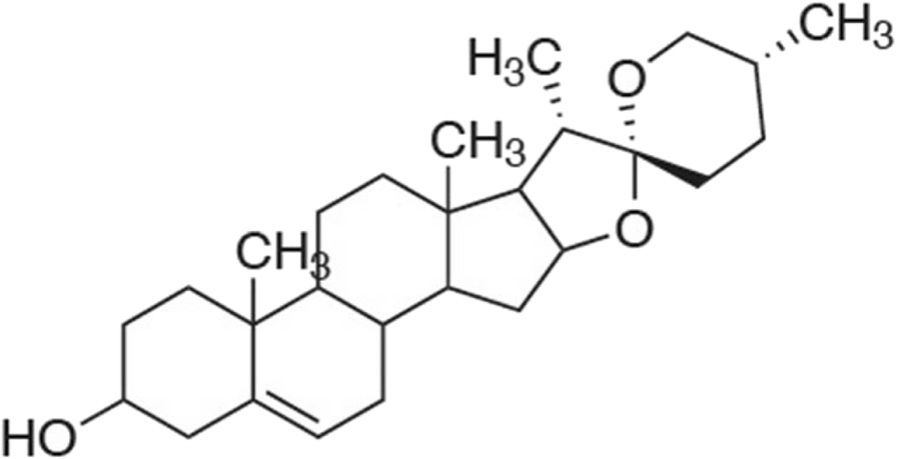
Chemical structure of diosgenin

*Costus speciosus* is used as food and medicine by the Kannikars, the primitive hill tribes of South India [1]. Recently it is used in drug industry as a natural source of diosgenin which is a steroidal sapogenin used for synthesis of sex hormones, cortisone and oral contraceptives [2]. Diosgenin content up to 3.37 % has been reported in rhizome of *C. speciosus* [3]. Diosgenin (Fig. 1), is a precursor of sex hormones (progesterone), corticosteroids (corticosone) and contraceptives [4]. Diosgenin induces apoptosis in cancerous cells by cyclooxygenase up -regulation and in HeLa cells by caspase pathway [5]. Dioscin, a derivative compound from Diosgenin, has been reported to induce apoptosis in HeLa cells through caspase −9 and caspase-3 pathway [6]. It causes an inhibition of growth of fibroblast –like synoviocytes in human rheumatoid arthritis with apoptosis induction associated with cylooxygenase-2 up-regulation [7]. Diosgenin has both antioxidant property and anti-cholesterolomic activity. It has been reported to have various effects such as hypocholesterolemic action in rat [8], antioxidant activity in HIV patients with dementia and apoptosis through cyclooxygenase activity in osteosarcoma cells [9]. Cholesterol-lowering activity of saponins, which was demonstrated in animal [10], and human trials were attributed to inhibition of the absorption of cholesterol from the small intestine, or the re -absorption of bile acids [11]. So, in the present study, we evaluated the anticancer and apoptotic effects on cell Proliferation activities of diosgenin isolated from *C. speciosus.*

## Methods

### Raw material

*C. speciosus* was procured from a fixed vendor in the local market of Al Jouf, KSA. Specimens were identified at the Aljouf University (Aljouf, Saudi Arabia) and voucher specimen (No. 13) was deposited at the Herbarium of the Department in the cited university. All cell maintaining materials was obtained from Cambrex, BioScience (Copenhagen, Denmark), while all other fine chemicals were from Sigma-Aldrich (CT, USA).

### Hydrolysis and HPTLC analysis

Dried tuber powder (5 g) was treated with slightly modified method described by Drapeau et al. [12]. Samples were hydrolyzed in 150 ml of refluxing 20 % H_2_SO_4_ in 70 % isopropanol for 8 h. The extract was filtered and extracted with hexane (50 ml × 3). The three hexane extracts were combined and rinsed thrice times with 5 % alkali and then rinsed thrice with distilled water. The extract was then passed through a column of Na_2_SO_4_ to eliminate any remaining water. The samples were concentrated to dryness by evaporating the solvent at 40 °C in a Rotary evaporator. The dried crude extract was solubilized in 0.5 ml of chloroform prior to the quantitative determination of diosgenin by thin layer chromatography as reported by Kshirsagar et al. [13].

### Cell culture for cancer cell lines

Hepatocellular carcinoma Hep G2 Cells, breast adenocarcinoma MCF-7 cells and colon carcinoma HCT-116 cells were purchased from ATCC, VA, USA. HepG2 and MCF-7 cells were cultured in RPMI medium, while HCT-116 cells were cultured in McCoy’s medium. Both were supplemented with 10 % fetal bovine serum, 2 μmol/ml L-glutamine, 250 ng/ml fungizone, 100 units/ml penicillin G sodium, and 100 units/ml streptomycin sulphate at 37 °C in a humidified 5 % CO_2_ incubator. The cultures were passaged every four days by trypsinization using 1 ml trypsin/EDTA solution for 5 min at 37 °C. Cells were used when confluence had reached 75 %. Trypsin/EDTA solution consists of 0.25 mM trypsin and 1 mM EDTA dissolved in phosphate buffer [14].

### Cell culture for immune cells

Lymphoblastic leukemia 1301 cells and raw murine macrophage (RAW 264.7) cell lines were purchased from ATCC, VA, USA. RAW 264.7 cells were grown in RPMI-1640. Media were supplemented with 10 % fetal bovine serum (FBS), 2 mM L-glutamine, containing 100 units/ml penicillin G sodium, 100 units/ml streptomycin sulphate, and 250 ng/ml amphotericin B. Cells were maintained at sub-confluency at 37 **°**C in humidified air containing 5 % CO_2_. For sub-culturing, monolayer cells were harvested by scraping. Cells were used when confluence had reached 75 % [14].

### Preparation of cell lysates for ELISA

After cells treatment as the experimental design, collected cells were washed and centrifuged for 10 min at 1000 × g. Cell pellets were lysed in 0.5 ml of ice-cold lysis buffer. This buffer consists of 50 mM Tris HCl, 150 mM NaCl, 1 mM EGTA, 1 mM EDTA, 20 mM NaF, 100 mM Na3VO4, 0.5 % NP40, 1 % Triton X-100, 1 mM phenylmethylsulfonyl fluoride, 10 mg/ml aprotinin, and 10 mg/ml leupeptin (pH 7.4). Cell lysates were passed through a 21-gauge needle to break up cell aggregates, and then centrifuged at 14,000 × g for 15 min at 4 °C [14].

### Anticancer assay

Cytotoxicity of tested sample against different cancer cell lines was measured using the MTT Cell Viability Assay. MTT (3-[4,5-dimethylthiazole-2-yl]-2,5-diphenyltetrazolium bromide) assay is based on the ability of active mitochondrial dehydrogenase enzyme of living cells to cleave the tetrazolium rings of the yellow MTT (5 mg/ml of MTT in 0.9 % NaCl) and form blue insoluble formazan crystals. Solubilization of the crystals by acidified isopropanol (0.04 N HCl in absolute isopropanol) was carried out. The extent of the reduction of MTT was quantified by measuring the absorbance at 570 nm [14]. The number of viable cells is directly proportional to the level of soluble formazan dark blue color.

Cells (0.5×10^5^ cells/well) in serum-free media were placed in a flat bottom 96-well microplate, and treated with 20 μl of different concentrations of the tested sample for 20 h at 37 °C, in a humidified 5 % CO_2_ atmosphere. After incubation for 24 h, cell was submitted to MTT assay. Triplicate repeats were performed for each concentration and the average was calculated. Data were expressed as the percentage of relative viability compared with the untreated cells compared with the vehicle control, with cytotoxicity indicated by <100 % relative viability. The percentage of relative viability was calculated using the following equation:

$$ \left[\mathbf{Absorbance}\ \mathbf{of}\ \mathbf{treated}\ \mathbf{cells}/\mathbf{Absorbance}\ \mathbf{of}\ \mathbf{control}\ \mathbf{cells}\right]\mathbf{X}\kern0.5em \mathbf{100} $$

The half maximal inhibitory concentration of cell viability (IC_50_) was calculated by from the linear equation of sample concentration and cell viability %.

### Cell death mode and mediators

#### Cell cycle analysis

MCF-7 cells (5×10^5^) were collected after different treatments, and then washed two times with PBS, resuspended in 300 μl of PBS, and fixed with 4 ml of ice-cold 70 % ethanol. The cells were centrifuged to remove ethanol and washed once in PBS. The cell pellets were then resuspended in 1 ml of a solution of 0.1 % Triton X-100 in PBS, 0.2 mg/ml RNase A, and 10 μg/ml propidium iodide (PI) and incubated for 30 min at room temperature. The stained cells were analyzed by flow cytometry [14].

#### Fluorescence analysis of apoptosis and necrosis in living cells

The mode of cell death was determined by investigating apoptosis and necrosis ratios using double stain with acridine orange/ethidium bromide according to the method of Ribble et al., [15]. Acridine orange/ethidium bromide (AO/EB, 100 μg/ml PBS) staining is used to visualize nuclear changes and apoptotic body formation that are characteristic of apoptosis. AO is a nucleic acid selective fluorescent cationic membrane-permeable dye that will stain all cells and makes the nuclei appears green. EB is a dye that is only taken up by cells when cytoplasmic membrane integrity is lost, to intercalate DNA staining the nucleus red. AO permeates all cells staining the nuclei green, where EB is only taken up by cells losing their cytoplasmic membrane integrity staining the nucleus red. Thus live cells have a normal green nucleus; early apoptotic cells have bright green to yellow nucleus with condensed or fragmented chromatin; late apoptotic cells display fragmented orange chromatin and cells have died from direct necrosis have a structurally dark orange to red nucleus. Cells were cultured in six well plates at a density of 5 × 10^5^ cells/well. Triplicate wells were treated with tested sample and incubated for 48 h at 37 °C in a humidified 5 % CO_2_ atmosphere. Finally cells were removed by trypsinization and resuspended in 50 μl PBS and 2 μl of a solution containing 100 μg/ml AO and 100 μg/ml EB in PBS was added. Stained cell suspension (10 μl) was placed on a clean microscope slide and covered with a coverslip. Cells were visualized and counted by fluorescence microscopy. For each sample, at least 500 cells were counted, and the percentage of apoptotic or necrotic cells was determined as:

$$ \%\ \mathrm{of}\ \mathrm{apoptotic}\ \mathrm{or}\ \mathrm{necrotic} = \left(\mathrm{the}\ \mathrm{total}\ \mathrm{number}\ \mathrm{of}\ \mathrm{apoptotic}\ \mathrm{or}\ \mathrm{necrotic}\ \mathrm{cells}/\ \mathrm{total}\ \mathrm{number}\ \mathrm{of}\ \mathrm{cells}\ \mathrm{counted}\right)\ \mathrm{x}\ 100. $$

#### Estimation of caspase-3 and death receptor-4

The levels of Caspase-3 and death receptor (DR-4) were measured in cell lysate by the quantitative indirect immunoassay ELISA technique, according to the method of Wang et al. [16]. The following reagents were prepared: Washing buffer (PBS/0.05 % polyoxyethylene-20; Tween-20); blocking buffer (PBS/0.05 % Tween-20/5 % FBS); diluents buffer (PBS/Tween-20/1 % FBS); and substrate buffer (equal volumes of 3,3',5,5'-tetramethyl benzidine [TMB; o.4 g/]) and H_2_O_2 [_0.02 % in citric acid buffer; KPL, Kirkegaard and Perry Lab. Gaithersburg, MD, USA]). Briefly, 96-wells bottom polystyrene microplates were coated with cell lysates (50 μl/well) and incubated for 1 h at 37 °C and then washed by washing buffer before incubated with blocking buffer for 90 min at 37 °C. The wells were washed and then antibodies (1 mg/ml Rabbit polyclonal to DR-4 or caspase-3, Abcam Inc., Cambridge, MA, USA) were added. After the incubation for 1 h at 37 °C, polyclonal Goat anti-rabbit peroxidase conjugate (1:2000, Jackson Immunsearch Lab, USA) was added to wells. After 1 h, wells were washed and the substrate buffer was applied and the color development was stopped by adding 1 M HCl. The intensity of the developed yellow color was measured at 450 nm using microplate reader (FLUOstar OPTIMA, BMG LABTECH GmbH, Offenburg, Germany). The level of DR-4 was expressed as optical densities values and compared with the activity of paclitaxel.

### Proliferation index of lymphocytes and macrophages

The effect of the tested sample proliferation index of lymphocytes and macrophages was investigated using the MTT Assay in lymphoblastic leukemia 1301 cells and raw murine macrophage (RAW 264.7). Cells (0.5×10^5^ cells/well) in serum-free media were placed in a flat bottom 96-well microplate, and treated with 20 μl of different concentrations of the tested sample. After incubation for 72 h, cells were submitted to MTT assay. The results were compared with *Echinacea purpurea* root extract as a known standard immunostimulant.

### Statistical analysis

Data were statistically analyzed using IBM computer supplied with Statistical Package for Social Scientists (SPSS) 10.00 for windows (SPSS Inc., Chicago, IL., USA). The student's unpaired *t*-test as was used to detect the statistical significance. P value was considered insignificant if more than 0.05.

## Results and discussion

Currently chemotherapy is regarded as one of the most efficient cancer treatment approach. Although chemotherapy significantly improves symptoms and the quality of life of patients with liver cancer, only modest increase in survival rate can be achieved. Faced with palliative care, many cancer patients use alternative medicines, including herbal therapies. Conventional chemotherapy remains ineffective in curing hepatocellular carcinoma due to its high hepatotoxicity. Several plant-based extracts have been shown to be effective in liver cancer therapy and prevention [17]. Given the several health promoting attributes to *C. speciosus*, the principle objective of the study was to evaluate and establish the anticancer efficacy of *C. speciosus* tuber extracts using human hepatocellular carcinoma cell lines. Previous work demonstrated in *C. speciosus* rhizome extract exhibited antioxidant and antiproliferative properties in human cancer cell lines [18]. Our study represents the first time to study anticancer activity, apoptotic and inhibitory effects on cell proliferation of diosgenin isolated from *C. speciosus*.

### Anticancer activity

**Fig. 2:**
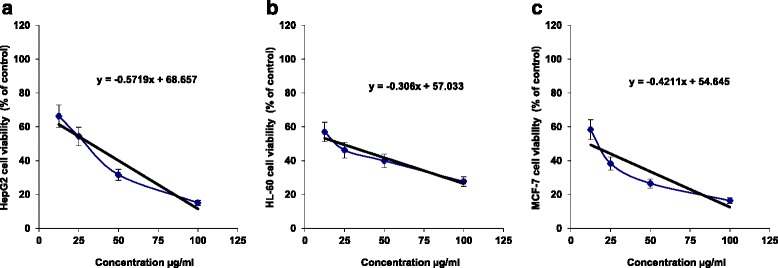
The cytotoxicity (% of control) of different concentrations (μg/ml) of the tested sample against (**a**) human HepG2, (**b**) human HL-60 and (**c**) human MCF-7 cells after 24 h of incubation as assayed by MTT. (*n* = 4)

Investigation of the anticancer activity of the tested sample was carried out using three different human cancer cell lines and assayed by a metabolic viability test (MTT). The results indicated that the treatment of HepG2 cells with the sample resulted in a cytotoxic effect as concluded from the IC_50_ value 32.62 μg/ml, as shown in Fig. 2a, while the treatment of HepG2 cells with paclitaxel, a known anti-cancer drug, resulted in an IC_50_ value of 0.48 μg/ml. In HL-60 Cells, the sample treatment resulted in moderate inhibition of the cell viability, as shown at Fig. 2b, and resulted in an IC_50_ value of 22.98 μg/ml, while the treatment of HCT-116 cells with paclitaxel resulted in an IC_50_ value of 0.78 μg/ml. The treatment of MCF-7 cells with the tested sample resulted in high inhibition in the cell viability, as shown at Fig. 2c, and resulted in an IC_50_ value of 11.03 μg/ml, while the treatment of MCF-7 cells with paclitaxel resulted in an IC_50_ value of 0.61 μg/ml.

Breast cancer is the most frequent cancer among women. Approximately 1.67 million new breast cancer cases were reported in 2012, which accounts for 25 % of all new diagnosed cancer [19]. The current treatment strategies for breast cancer patients such as surgery, chemotherapy and radiotherapy or the combination of radiotherapy and chemotherapy have successfully increased the five-year survival rate of the breast cancer patients. Nevertheless, the long-term survival remains poor due to cancer relapse and metastasis. In the present study, diosgenin exhibited strong cytotoxic properties against caspase-3 deficient MCF-7 cells (Fig. 2c). According to the National Cancer Institute guidelines, diosgenin is a great candidate to be developed as an anticancer agent for breast cancer as the IC_50_ value was less than 11.03 μg/mL towards MCF-7 cells after treatment for 72 h [20]. Diosgenin treatment on MCF-7 cell lines showed significant decrease in growth rate compared with control. On the other hand the percentage of non-viable cells on cell lines increased with the increasing period of treatment. These results were in concordance with the studies investigated the cytotoxic effect of Goniothalamin towards human breast cancer cells by Al- Qubaisi et al. [21]. Hence present study shows the efficacy of diosgenin for the cytotoxicity towards MCF-7 cells thus suggesting protection against breast cancer.

### Cell cycle analysis

**Fig. 3:**
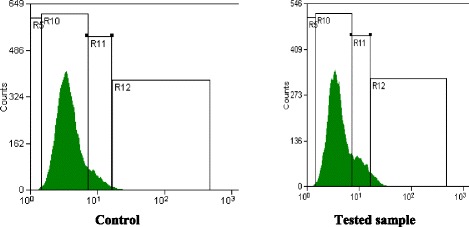
Cell cycle analysis of MCF-7 cells: DNA frequency distribution of cells cultured in control (*left*), Sample-treated (*right*), in one representative capture

In order to explain the anti-cancer property of the tested sample in MCF-7 cells, we investigated their role in cell cycle progression. As shown in Fig. 3, treatment of cells with tested sample (30 % of the IC_50_) resulted in a predominated growth arrest at the S- and G2/M-phases (*P* < 0.01), where the large cell population at S-phase represents a considerable delay in the progression in that phase, as compared with control cells. No evident sub-G0/G1 peak was noted after the sample.

### Fluorescence analysis of apoptosis and necrosis in living cells

**Fig. 4:**
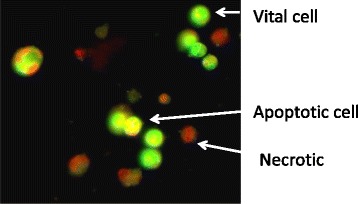
Representative images for the mode of cell death in the sample-treated MCF-7 cells (50 % of IC50), as stained by AO/EB and captured under fluorescence microscope (x200). Vital cells (green), early apoptotic (bright green to yellow), late apoptotic (orange) and necrotic cells (red)

**Fig. 5:**
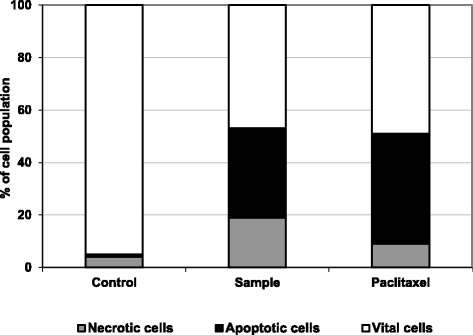
The cell population percentage of vital (white segments), apoptotic (black segment) and necrotic (gray segments) cells were counted after treatment with the tested sample and paclitaxel. Results represent the mean of three independent experiments

Acridine orange (AO) stained both live and dead cells, while ethidium bromide (EB) stained only cells with lost membrane integrity. Living cells will appear green, while early apoptotic cells are containing bright green to yellow dots in the nuclei due to chromatin condensation and nuclear fragmentation. Late apoptotic cells also have EB (orange), but unlike necrotic cells, the late apoptotic cells exhibited condensed and fragmented nuclei. Necrotic cells are in deep orange to red, but have a nuclear morphology resembling that of living cells, with no condensed chromatin. The percentages of vital, apoptotic and necrotic cells were determined and compared with untreated cells, as demonstrated in Fig. 4. As shown in Fig. 5, MCF-7 cells treated with the tested sample and paclitaxel, using half IC_50_ concentration of each, showed a significant (*P* < 0.05) increase in the total percentage of cell death into >50 %, where there was a significant increase in both of apoptotic and necrotic cell populations, however the majority population in dead cells in the tested sample and paclitaxel was apoptotic population.

### Estimation of caspase-3 and death receptor 4

**Fig. 6:**
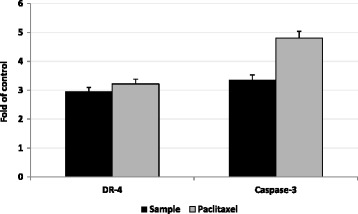
The effect of treatment with the tested sample (black bars, 50 % of IC50) on apoptotic cell death pathway in MCF-7 cells compared with paclitaxel activity (gray bars). DR4 and caspase-3 levels in treated cells were compared with control cells. The data represented as number of folds in relation to control absorbance readings, which were 7.3, and 6.5 milliabsorbance, respectively

Apoptosis can be started through two distinct pathways, the death receptor (extrinsic pathway) mitochondrial mediated (intrinsic pathway). Both lead to activation of the caspase cascades "executioner caspases" such as caspase-3, −6 and −7. The active executioner caspases bind cellular substrates, leading to the characteristic biochemical and morphological changes observed in dying cells. This is followed by chromatin condensation, nuclear shrinkage and DNA fragmentation. Binding of cytoskeletal proteins leads to cell fragmentation and formation of apoptotic bodies [22, 23]. As shown in Fig. 6, the levels of DR4 and caspase-3 were significantly increased (*P* < 0.01) in MCF-7 cells treated with the tested sample compared to untreated cells and possessed a similar activity of paclitaxel in DR4 induction but lower induction in caspase-3.

### Proliferation index of lymphocytes and macrophages

**Fig. 7:**
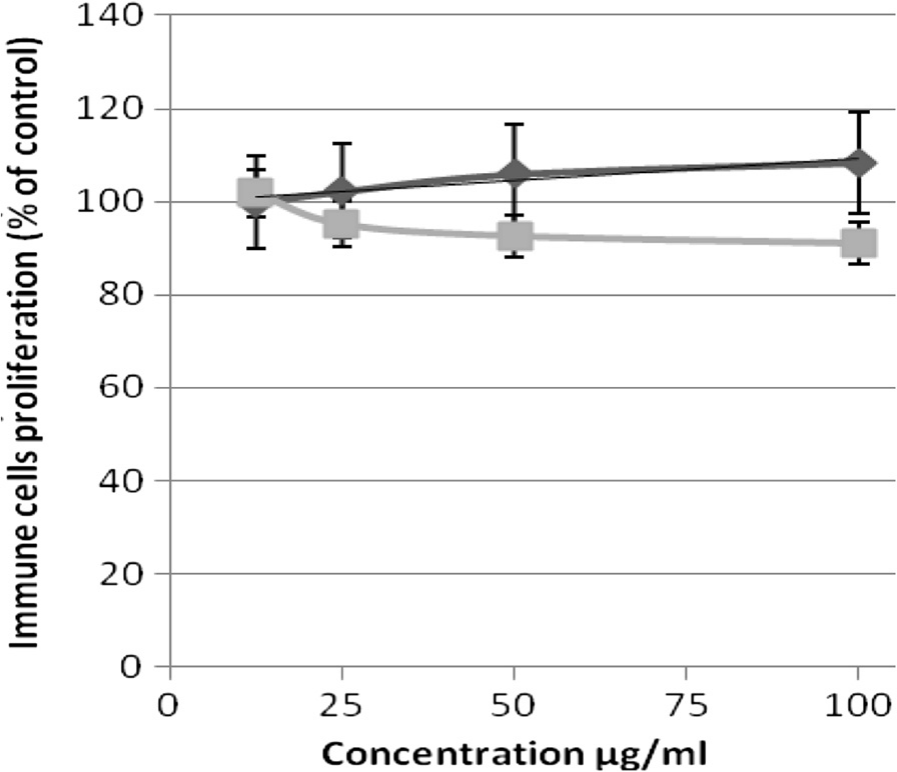
The proliferation (% of control) of different concentrations (μg/ml) of the tested sample in lymphoblastic leukemia 1301 cells (grey line) and Raw murine macrophage RAW 264.7 (black line) after 72 h of incubation as assayed by MTT. (*n* = 4)

Investigation of the effect of the tested sample on the proliferation of the immune cells was carried out using two different immune cell lines and assayed by a MTT test. The results indicated that the treatment of macrophages and lymphocytes with different sample concentrations resulted in no change in the proliferation index compared to the control untreated cells, as shown in Fig. 7. On the other hand the treatment of macrophages or lymphocytes with diosgenin (250 μg/ml) resulted in an induction in the cell proliferation up to 3.2-fold and 2.1-fold of control, respectively.

## Conclusion

In conclusion, our current study is the first to identify the remarkable anticancer activity of, apoptotic and inhibitory effects on cell proliferation of diosgenin isolated from *C. speciosus*. Our data generate compelling evidence for further evaluation of this constituent as chemo preventive regimen for liver cancer. These studies are valuable for identifying plant-based chemotherapeutic drugs as currently used liver cancer drug causes many side effects. However, animal and human studies are indeed required to prove the safety and efficacy of application of diosgenin of *C. speciosus* as a potential chemo preventive agent in clinical practice.

## References

[1] Ammal JEK, Prasad N (1984). Ethnobotanical finding on *Costus speciosus* (Koen) sm. Among the Kannikkars of Tamil Nadu. J Econ Tax Bot.

[2] Pawar VA, Pawar PR (2014). *Costus speciosus*: An Important Medicinal Plant. International Journal of Science and Research.

[3] Singh I, Gautam Y, Vimala Y (2013). Detection and isolation of diosgenin from *Costus speciosus* callus raised from non-germinal seeds. Int J Chem and Life Sciences.

[4] Coursey DG (1967). Yams an account of the nature, origin cultivation and utilization of the useful members of the Diocoreaecaea.

[5] Huo R, Zhou Q, Wang B, Tashiro S, Onodera S, Ikejima T (2004). Diosgenin induces apoptosis in HeLa cells via activation of caspase pathway. Acta Pharmacologica Sinica.

[6] Cai J, Liu M, Wang Z, Ju Y (2002). Apoptosis induced by dioscin in HeLa cells. Biol Pharm Bull.

[7] Liagre B, Pascale V, Cecile C, Chaissoux LJ, Beneytout LJ (2004). Diosgenin, a plant steroid, induces apoptosis in human rheumatoid arthritis synovyocytes, with cyclohexogenase −2 overexpression. Arthritis Res Ther.

[8] Accatino L, Pizzaro M, Solis N, Koenig CS (1998). Effects of diosgenin a plant –derived steroid, on bile secretion and hepatocellular cholestasis induced by estrogens in the rat. Hepatology.

[9] Moalic S, Liagre B, Corbiere C, Bianchi A, Dauca M, Bordji K, Beneytout JL (2001). A plant steroid, diosgenin induces apoptosis, cell cycle arrest and COX activity in osteosarcoma cells. FEBS Lett.

[10] Matsuura M (2001). Saponins in garlic as modifiers of the risk of cardiovascular disease. J Nutr.

[11] Oakenfull D, Sidhu GS (1990). Could saponins be a useful treatment for hypercholesterolemia?. Eur J Clin Nutr.

[12] Drapeau D, Sauvaire Y, Blanch HW, Wilke CR (1986). Improvement of diosgenin yield from *Dioscorea deltoidea* plant cell culture by use of a non-traditional hydrolysis method. Planta Med.

[13] Kshirsagar VB, Deokate UA, Bharkad VB, Khadabadi SS (2008). HPTLC method development and validation for the simultaneous estimation of diosgenin and Levodopa in marketed formulation. Asian J Research Chem.

[14] Hansen MB, Nielsen SE, Berg K (1989). Re-examination and further development of a precise and rapid dye method for measuring cell growth/cell kill. J Immunol Methods.

[15] Ribble D, Goldstein NB, Norris DA, Shellman YG (2005). A simple technique for quantifying apoptosis in 96-well plates. BMC Biotechnol.

[16] Wang Y, Yang H, Liu H, Huang J, Song X (2009). Effect of staurosporine on the mobility and invasiveness of lung adenocarcinoma A549 cells: an in vitro study. BMC Cancer.

[17] Stagos D, Amougias GD, Matakos A, Spyrou A, Tsatsakis AM, Kouretas D (2012). Chemoprevention of liver cancer by plant polyphenols. Food and Chemical Toxicology.

[18] Baskar AA, AlNumair KS, Alsaif MA, Ignacimuthu S (2012). In vitro antioxidant and antiproliferative potential of medicinal plants used in traditional Indian medicine to treat cancer. redox Report.

[19] GLOBOCAN. Breast cancer: estimated incidence, mortality and prevalence worldwide in 2012. International Agency for Research on Cancer, World Health Organization. http://globocan.iarc.fr/Pages/fact_sheets_cancer.aspx

[20] Boyed MR, Teicher B (1997). The NCI *in vitro* Anticancer Drug Discovery Screen. Anticancer Drug Development Guide; Preclinical Screening, Clinical Trials and Approval.

[21] Al-Qubaisi M, Rozita R, Yeap SK, Omar AR, Ali AM, Alitheen NB (2011). Selective cytotoxicity of Goniothalamin against Hepatoblastoma HepG2 Cells. Molecules.

[22] Estaquier J, Vallette F, Vayssiere JL, Mignotte B (2012). The mitochondrial pathways of apoptosis. Adv Exp Med Biol.

[23] Krysko DV, Vandenabeele P (2008). From regulation of dying cell engulfment to development of anti-cancer therapy. Cell Death Differ.

